# Low Vitamin D Levels Are Associated with Increased Cardiac Iron Uptake in Beta-Thalassemia Major

**DOI:** 10.3390/diagnostics13243656

**Published:** 2023-12-13

**Authors:** Antonella Meloni, Laura Pistoia, Cristina Vassalle, Anna Spasiano, Ilaria Fotzi, Sergio Bagnato, Maria Caterina Putti, Antonella Cossu, Francesco Massei, Piera Giovangrossi, Silvia Maffei, Vincenzo Positano, Filippo Cademartiri

**Affiliations:** 1 Bioengineering Unit, Fondazione G. Monasterio CNR—Regione Toscana, 56124 Pisa, Italy; antonella.meloni@ftgm.it (A.M.); positano@ftgm.it (V.P.); 2 Department of Radiology, Fondazione G. Monasterio CNR—Regione Toscana, 56124 Pisa, Italy; laura.pistoia@ftgm.it; 3 Unità Operativa Complessa Ricerca Clinica, Fondazione G. Monasterio CNR—Regione Toscana, 56124 Pisa, Italy; 4 Medicina di Laboratorio, Fondazione G. Monasterio CNR—Regione Toscana, 56124 Pisa, Italy; cristina.vassalle@ftgm.it; 5 Unità Operativa Semplice Dipartimentale Malattie Rare del Globulo Rosso, Azienda Ospedaliera di Rilievo Nazionale “A. Cardarelli”, 80131 Napoli, Italy; spasiano.anna@tiscali.it; 6 Oncologia, Ematologia e Trapianto di Cellule Staminali Emopoietiche, Meyer Children’s Hospital IRCCS, 50139 Firenze, Italy; ilaria.fotzi@meyer.it; 7 Ematologia Microcitemia, Ospedale San Giovanni di Dio—ASP Crotone, 88900 Crotone, Italy; sergio249@alice.it; 8 Dipartimento della Salute della Donna e del Bambino, Clinica di Emato-Oncologia Pediatrica, Azienda Ospedaliero Università di Padova, 35128 Padova, Italy; mariacaterina.putti@unipd.it; 9 Ambulatorio Trasfusionale—Servizio Immunoematologia e Medicina Trasfusionale Dipartimento dei Servizi, Presidio Ospedaliero “San Francesco”, 08100 Nuoro, Italy; a.cossu.1@aslnuoro.it; 10 Unità Operativa Oncoematologia Pediatrica, Azienda Ospedaliero Universitaria Pisana—Stabilimento S. Chiara, 56126 Pisa, Italy; f.massei@med.unipi.it; 11 Servizio di Immunoematologia e Medicina Trasfusionale, Ospedale S. M. Goretti, 04100 Latina, Italy; p.giovangrossi@ausl.latina.it; 12 Cardiovascular and Gynaecological Endocrinology Unit, Fondazione G. Monasterio CNR—Regione Toscana, 56124 Pisa, Italy; silvia.maffei@ftgm.it

**Keywords:** thalassemia major, myocardial iron overload, vitamin D, parathyroid hormone

## Abstract

We evaluated the association of vitamin D and parathormone (PTH) levels with cardiac iron and function in beta-thalassemia major (β-TM) patients. Two-hundred and seventy-eight TM patients (39.04 ± 8.58 years, 56.8% females) underwent magnetic resonance imaging for the assessment of iron overload (T2* technique), biventricular function parameters (cine images), and replacement myocardial fibrosis (late gadolinium enhancement technique). Vitamin D levels were deficient (<20 ng/dL) in 107 (38.5%) patients, insufficient (20–30 ng/dL) in 96 (34.5%) patients, and sufficient (≥30 ng/dL) in 75 (27.0%) patients. Deficient vitamin D patients had a significantly higher frequency of myocardial iron overload (MIO; global heart T2* < 20 ms) than patients with sufficient and insufficient vitamin D levels and a significantly higher left ventricular end-diastolic volume index and mass index than patients with sufficient vitamin D levels. PTH was not associated with cardiac iron, function, or fibrosis. In the multivariate regression analysis, vitamin D, serum ferritin, and pancreatic iron levels were the strongest predictors of global heart T2* values. In receiver operating characteristic curve analysis, a vitamin D level ≤ 17.3 ng/dL predicted MIO with a sensitivity of 81.5% and a specificity of 75.3% (*p* < 0.0001). In TM, the periodic and regular assessment of vitamin D levels can be beneficial for the prevention of cardiac iron accumulation and subsequent overt dysfunction.

## 1. Introduction

Thalassemia is a group of inherited blood disorders caused by mutations in the genes responsible for producing hemoglobin’s alpha- and beta-globin chains [1]. Beta-thalassemia major (β-TM) (also known as Cooley’s anemia) is the most severe form of beta-thalassemia, and it is characterized by strongly impaired or absent production of functional beta-globin chains, with a consequent excess of alpha-globin chains [2,3]. These excess alpha-globin chains tend to aggregate and form insoluble structures within the red blood cells, making them less flexible and more prone to damage, contributing to their premature destruction (hemolysis) [4]. The combination of inefficient hemoglobin production, hemolysis, and ineffective erythropoiesis (i.e., destruction of red blood cell precursors in the bone marrow) results in severe anemia [5]. Lifelong regular blood transfusions and iron chelation are the cornerstones of disease management [6,7]. The introduction of routine transfusion therapy for the correction of anemia 60 years ago transformed β-TM from a fatal childhood illness into a chronic disorder. However, since the body has no natural mechanism for excreting excess iron [8,9], regular blood transfusions can cause iron accumulation in the body, which can damage organs such as the heart, liver, and pancreas [10,11,12]. The aim of chelation therapy is to maintain the body’s iron at safe levels at all times by preventing iron accumulation or eliminating the already-occurring iron deposition [13,14,15,16]. Proper monitoring of tissue iron levels is vital for the success of chelation therapy, offering the possibility to select the most appropriate chelating agent based on the individual patient’s needs and to assess the response rate to the chosen chelation regimen. Magnetic resonance imaging using the T2* technique is a perfect fit for this purpose [17,18,19,20,21,22,23]. Paving the way for the robust, reproducible, and non-invasive assessment of iron distribution in all organs, MRI has significantly impacted the management of TM patients [24,25,26,27].

Although the improvements in management and treatment have significantly improved the survival of TM patients [28,29], iron-induced cardiomyopathy still remains the main cause of mortality among TM patients [29,30]. Iron accumulates initially in the ventricular myocardium and subsequently in the atrial myocardium [31]. Ventricular iron deposition causes early diastolic dysfunction featuring a pseudo-normalized or restrictive filling pattern, while the advanced-stage disease is characterized by dilated cardiomyopathy with systolic impairment [31,32]. To stop the progression of early disease and prevent overt cardiovascular disease, it is important to address the early iron accumulation in the myocardium and identify those factors that can contribute to an increased risk of cardiac iron loading.

In a small study involving 24 thalassemia major patients, Wood et al. demonstrated an association between vitamin D deficiency and cardiac iron uptake [33]. The mechanism behind this link is that decreased vitamin D levels stimulate the expression of the transmembrane L-type voltage-dependent calcium channels (LVDCCs) [34]. These channels are primarily used to transport calcium but play an important role as a portal for the uptake of non-transferrin-bound iron (NTBI) into the myocardium [35,36]. The long-term accumulation of NTBI and the resulting increase in the labile iron pool can have detrimental effects on cellular function due to reactive oxygen species generation, which, in turn, can lead to cellular dysfunction, apoptosis, and necrosis [37,38]. Indeed, studies conducted in iron-loaded mice demonstrated that administering L-type calcium channel antagonists, such as nifedipine, amlodipine, and verapamil, could inhibit cardiac iron uptake and decrease oxidative stress [35,39]. Other studies involving relatively small cohorts of TM patients did not find a significant correlation between vitamin D and cardiac iron levels [40,41]. Importantly, a Greek study identified increased parathyroid hormone (PTH) levels as a strong predictor of enhanced cardiac iron content [40]. Vitamin D deficiency increases PTH production, and secondary hyperparathyroidism can further intensify iron uptake into cardiomyocytes through the LVDCC. However, the data on the link between PTH and cardiac iron are few and controversial [40,41].

Besides the heart, the pancreas, selectively or near selectively, loads circulating NTBI through the LVDCC [36]. Consequently, there is a strong link between the iron burden in these two organs, with a normal global pancreas T2* value showing a 100% negative predictive value for cardiac iron [42,43,44]. As far as we know, no studies have systematically investigated the correlation of vitamin and PTH levels with pancreatic iron in β-TM.

The aims of this multicenter study were to systematically explore the cross-sectional association of vitamin D and PTH levels with pancreatic and cardiac iron burden and with biventricular function parameters and to identify the strongest determinants of myocardial iron overload in a relatively large cohort of well-treated β-TM patients.

## 2. Materials and Methods

### 2.1. Study Population

The Extension-Myocardial Iron Overload in Thalassemia (E-MIOT) project is an Italian Network consisting of 66 thalassemia centers and 15 validated magnetic resonance imaging (MRI) sites [45], linked by a web-based database, collecting all clinical, laboratory, instrumental, and anamnestic data. The inclusion criteria of the E-MIOT project are as follows: (1) individuals of both genders, spanning all age groups, who have been diagnosed with thalassemia or sickle cell disease necessitating the measurement of organ iron content via MRI; (2) written informed consent to participation in the study; (3) written consent for the use or disclosure of protected health information; and (4) absence of contraindications to the MRI procedure.

Between May 2017 and November 2021, all adult β-TM patients attending the reference MRI center of the E-MIOT Network (Fondazione G. Monasterio CNR-Regione Toscana (FTGM), Pisa, Italy) had the opportunity to be part of a project funded by the Italian Ministry of Health and Tuscany Region, aimed at evaluating the bone health status in TM. According to this project, each patient had to undergo, on the same day of the MRI, a blood test for the measurement of different parameters of bone turnover, including vitamin D and PTH, and a dual X-ray absorptiometry (DEXA) for the assessment of bone mineral density. 

The two-hundred and seventy-eight β-TM patients consecutively enrolled in the E-MIOT project also participated in the bone-focused project.

Both E-MIOT and the Italian Ministerial projects received approval from the Ethics Committee of Area Vasta Nord Ovest (CEAVNO). The study was conducted per the principles of the Declaration of Helsinki. Written informed consent was obtained from all patients before enrollment. 

### 2.2. Biochemical Analysis

Pre-transfusion hemoglobin, ferritin, and liver function parameters, such as alanine aminotransferase (ALT), aspartate aminotransferase (AST), and gamma-glutamyl transferase (GGT), were assessed at the thalassemia centers where the patients were treated. These parameters were determined using commercially available kits at least four times per year, and the mean value obtained from these multiple measurements was used to represent a single value for each patient.

For the measurement of parathyroid hormone [1-84 PTH] and serum vitamin D [25-hydroxy vitamin D3 or 25(OH)D], blood samples were collected after 8 h of fasting. They were analyzed in the Medicine Laboratory of FTGM using a chemiluminescent immunoassay (CLIA) (LIAISON Assay, DiaSorin, Stillwater, MN, USA).

### 2.3. Magnetic Resonance Imaging

MRI scanning was performed within one week before a regularly scheduled blood transfusion using a 1.5 T scanner (Signa Excite or Artist, GE Healthcare, Milwaukee, WI, USA). Acquisitions were performed with breath-holding and ECG gating by using a 30-element cardiac phased-array receiver surface coil.

For the quantification of iron overload, a mid-transverse hepatic slice [46], five or more axial slices covering the whole pancreas [47], and basal, medium, and apical short-axis views of the left ventricle (LV) [48] were acquired with T2* multiecho gradient-echo sequences (10 echo times—TEs with an echo spacing of 2.26 ms). T2* image analysis was performed by expert operators using previously validated, custom-written software (HIPPO MIOT^®^, Version 2.0, Consiglio Nazionale delle Ricerche and Fondazione Toscana Gabriele Monasterio, Pisa, Italy, Year 2015) [49]. A circular region of interest (ROI) was drawn in an area of homogeneous hepatic tissue, avoiding blood vessels and other sources of artifacts [46]. Three ROIs were manually defined over the pancreatic head, body, and tail, encompassing parenchymal tissue and avoiding large blood vessels or ducts and regions affected by susceptibility artifacts arising from gastric or colic intraluminal gas [50]. The mean value of the signal intensity along all TE values was calculated for each ROI. The averaged decay curve was fit to a single exponential with a constant offset model. Liver iron concentration (LIC) values were derived from hepatic T2* values [51]. Global pancreatic T2* value was computed as the mean of T2* values from the three regions. According to the American Heart Association standardized segmentation, the myocardial T2* distribution was mapped into a 16-segment LV model (6 equiangular segments in basal and medium slices and 4 equiangular segments in the apical slice) [52]. The mean value of the signal intensity along all the TEs was computed for each segment, and the obtained decay curve was fit to the single exponential model. A truncation model was employed in heavily iron-overloaded hearts, eliminating the late points with a reduced signal-to-noise ratio [53]. An appropriate correction map was used to correct for susceptibility artifacts [49]. The global heart T2* value was the mean of all segmental values. 

To quantify biventricular size and function, steady-state free procession cine images were acquired in sequential short-axis slices (slice thickness 8 mm, no gap) from the atrio-ventricular ring to the apex [54]. Thirty cardiac phases were obtained per heartbeat. Manual post-processing was performed by expert operators using a commercially available software system (cmr42, Circle Cardiovascular Imaging Inc., Calgary, AB, Canada). Biventricular volumetry was performed by manual delineation of endocardial and epicardial contours in end-diastolic and end-systolic phases in each slice. The papillary muscles were manually outlined and were treated as myocardial mass rather than being classified as part of the blood pool. End-diastolic and end-systolic volumes (EDVs and ESVs, respectively) were determined by applying Simpson’s rule without geometrical assumptions about the ventricle shape. Ejection fraction (EF) was calculated applying the formula (EDV − ESV) × 100/EDV. The LV mass was calculated by multiplying the volume of the myocardium by its specific weight of 1.05 g/cm^3^.

To detect replacement/focal myocardial fibrosis, late gadolinium enhancement (LGE) short-axis and vertical, horizontal, and oblique long-axis images were obtained with a fast gradient-echo inversion recovery sequence 10–18 min after the intravenous administration of Gadobutrol (Gadovist^®^; Bayer, Berlin, Germany) at the standard dose of 0.2 mmoL/kg of body weight. LGE imaging was not performed in patients with a glomerular filtration rate < 30 mL/min/1.73 m^2^ and in patients who refused the contrast medium administration. LGE was considered present when visualized in two different views [55]. 

### 2.4. Diagnostic Criteria

The criteria for the classification of vitamin D status of the United States Endocrine Society were used for categorization. 25(OH)D levels lower than 20 ng/mL (<50 nmol/L) defined vitamin D deficiency, 25(OH)D levels between 20 and 29 ng/mL (50–75 nmol/L) defined vitamin D insufficiency, and 25(OH)D levels greater or equal to 30 ng/mL (≥75 nmol/L) [56] defined vitamin D sufficiency.

The normal PTH range was 4.4–58.6 pg/mL.

An MRI LIC ≥ 3 mg/g dry weight (dw) indicated significant hepatic iron load [57]. The lower cut-off for normal pancreatic T2* values was 26 ms [47]. A T2* measurement > 20 ms represented the “conservative” normal value for segmental and global heart T2* values [49,58].

### 2.5. Statistical Analysis

SPSS version 27.0 (IBM Corp, Armonk, NY, USA) and MedCalc version 19.8 (MedCalc Software Ltd., Ostend, Belgium) statistical packages were employed for statistical data analysis.

Continuous variables were presented as mean ± standard deviation (SD), while categorical variables were described using frequencies and percentages.

The Kolmogorov–Smirnov test was employed to evaluate the normality of distribution of quantitative variables.

Correlation analysis was conducted employing Pearson’s or Spearman’s tests where appropriate.

For continuous variables that followed a normal distribution, group comparisons were conducted using an independent-sample *t*-test (2 groups) or one-way analysis of variance (ANOVA) (>2 groups). Wilcoxon’s signed-rank or Kruskal–Wallis tests were utilized for continuous values with non-normal distribution. χ^2^ testing was used to compare frequencies and categorical variables. A Bonferroni post hoc test was carried out for pair-wise comparison within the groups.

Logistic regression was employed to assess the odds ratio (OR) along with 95% confidence intervals (CIs). The OR was used to estimate relative risk associated with dichotomous risk factors.

Univariate and stepwise multivariate regression analyses were conducted to identify determinants of global heart T2* values. Only those variables that exhibited a significance level of *p* < 0.05 in univariate regression analyses were incorporated into the multivariate regression analysis. Collinearity among variables included in the multivariate model was evaluated through the variance inflation factor (inflated if >5) and the tolerance statistics (inflated if <0.20).

Receiver operating characteristic (ROC) analysis was conducted to assess the diagnostic utility of the clinical factors, and the results were reported as areas under the curve (AUCs) with corresponding 95% CIs. The optimal cut-off value was determined using the Youden index method. 

A 2-tailed probability value ≤ 0.05 was used as the criterion for statistical significance in all tests. 

## 3. Results

### 3.1. Patient Characteristics 

All 278 patients were white. Patients were well balanced in terms of gender (56.8% women) and had a mean age of 39.04 ± 8.58 years (range: 18–68 years). All patients had started regular transfusions in early childhood to maintain a pre-transfusion hemoglobin concentration above 9–10 g/dL. All patients were under chelation therapy, started at a mean age of 4.41 ± 4.69 years. Specifically, patients born before the mid-to-late 1970s began chelation therapy in that period, whereas those born after the 1970s started receiving chelation therapy in early childhood.

The demographic, clinical, instrumental, and laboratory characteristics of the patients are summarized in Table 1.

MRI LIC values were inversely correlated with both global pancreas T2* values (R = −0.389; *p* < 0.0001) and global heart T2* values (R = −0.393; *p* < 0.0001), and a significant correlation was present between global pancreas and heart T2* values (R = 0.396; *p* < 0.0001). 

The contrast medium was administered in 191 (68.7%) patients, and replacement myocardial fibrosis was detected in 77 (40.3%). Among the patients with LGE areas, 77.9% had two or more foci of fibrosis, and the septal region was involved in 87.0% of the cases.

### 3.2. Correlates of Vitamin D Levels 

Mean vitamin D levels were 23.73 ± 10.90 ng/dL. 

Overall, 63.2% of patients received vitamin D supplementation. Patients who received vitamin D supplementation had significantly higher vitamin D levels than patients who did not receive vitamin D supplementation (24.41 ± 11.46 ng/mL vs. 20.90 ± 9.29 ng/mL; *p* = 0.024), while no difference between the two groups was found in MRI LIC values (8.48 ± 13.37 mg/g dw vs. 6.82 ± 8.46 mg/g dw; *p* = 0.686), global pancreas T2* values (10.26 ± 8.29 ms vs. 10.85 ± 8.77 ms; *p* = 0.925), or global heart T2* values (36.72 ± 9.97 ms vs. 36.19 ± 11.62 ms; *p* = 0.806).

A total of 75 (27.0%) patients were vitamin D-sufficient, 96 (34.5%) patients were vitamin D-insufficient, and 107 (38.5%) patients were vitamin D-deficient. Table 2 compares demographic, clinical, instrumental, and laboratory characteristics among the three groups identified based on their vitamin D levels. 

Patients with deficient vitamin D levels were significantly younger than patients with adequate or insufficient vitamin D levels (*p* < 0.0001 and *p* = 0.042, respectively), while no difference was detected in the male-to-female ratio. Body mass index was not associated with vitamin D levels. Mean pre-transfusion hemoglobin was comparable among the three groups, while patients deficient in vitamin D exhibited significantly higher mean ferritin, ALT, and AST levels than patients sufficient in vitamin D (*p* = 0.030). 

**Figure 1:**
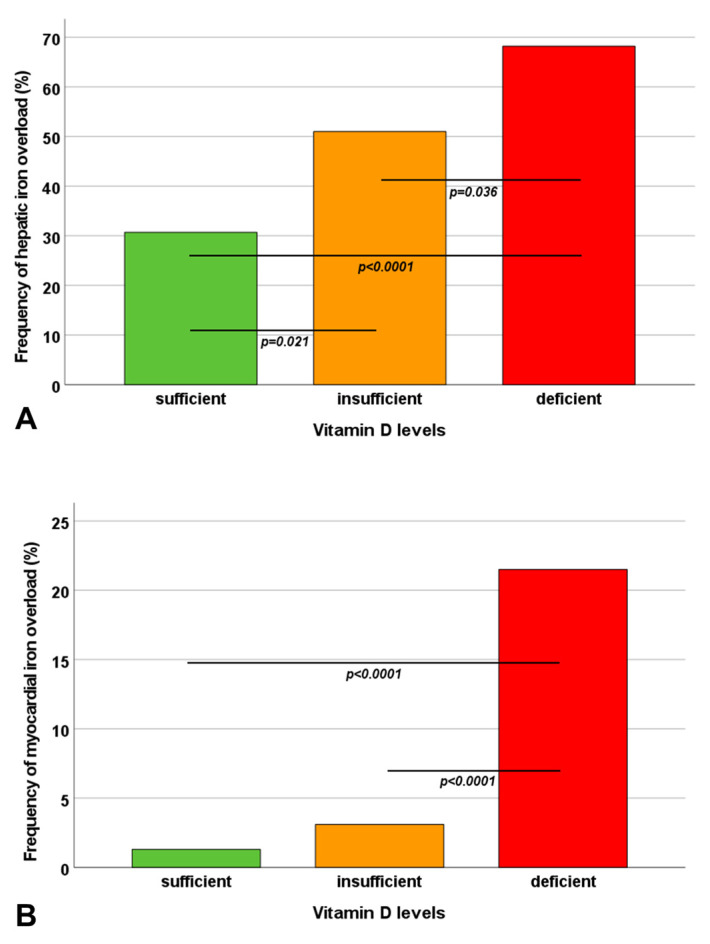
Frequency of hepatic iron overload (**A**) and myocardial iron overload (**B**) in the three groups identified based on vitamin D levels. The horizontal lines indicate a significant difference between two groups.

A significant inverse correlation was detected between MRI LIC values and vitamin D levels (R = −0.334; *p* < 0.0001). MRI LIC values were significantly higher in vitamin D-deficient patients than in patients with insufficient and normal vitamin D levels (*p* < 0.0001 and *p* = 0.009, respectively) and in patients with insufficient versus normal vitamin D levels (*p* = 0.006). A significant difference among the three groups was found regarding the prevalence of hepatic iron overload (Figure 1A). 

According to the logistic regression analysis, an increased serum ferritin level (>2500 ng/L) was not associated with a significantly increased risk of inadequate vitamin D levels (<30 ng/mL) (OR = 6.37, 95%CI = 0.81–50.10; *p* = 0.078), while hepatic iron overload emerged as a significant risk factor for inadequate vitamin D levels (OR = 3.41, 95%CI = 1.93–5.99; *p* < 0.0001). 

Vitamin D levels were significantly correlated with global pancreas T2* values (R = 0.277; *p* < 0.0001). A significant difference in global pancreas T2* values was found between patients with normal and deficient vitamin D levels (*p* < 0.0001), but the frequency of pancreatic iron overload was comparable among the three groups.

Vitamin D levels were significantly correlated with global heart T2* values (R = 0.287; *p* < 0.0001). Vitamin D-deficient patients exhibited significantly lower global heart T2* values than patients with normal and insufficient vitamin D levels (*p* < 0.0001 and *p* = 0.015, respectively). A significant difference among the three groups was found in terms of the prevalence of myocardial iron overload (Figure 1B). Patients with deficient vitamin D levels had a significantly higher risk of myocardial iron overload than patients with adequate vitamin D levels (OR = 20.62, 95%CI = 2.67–153.72; *p* = 0.004) and patients with insufficient vitamin D levels (OR = 8.49, 95%CI = 2.46–29.29; *p* = 0.001). The number of cardiac segments with T2* < 20 ms was significantly increased in vitamin D-deficient patients compared to patients with normal as well as insufficient vitamin D levels (*p* < 0.0001 for both comparisons). 

Patients with deficient vitamin levels showed a significantly higher left ventricular (LV) end-diastolic volume index (*p* = 0.003) and LV mass index (*p* = 0.006) when compared to patients with normal vitamin D levels. LV and right ventricular (RV) ejection fractions were lower in patients with vitamin D deficiency, although no statistical difference with respect to the other groups was found.

The frequency of replacement myocardial fibrosis was comparable among the three groups.

### 3.3. Correlates of PTH Levels 

Mean PTH levels were 17.13 ± 7.66 pg/mL. No patient showed increased PTH levels, while four patients had subnormal PTH levels.

PTH levels were comparable between males and females (16.18 ± 6.79 pg/mL vs. 17.85 ± 8.21 pg/mL; *p* = 0.062) but were inversely correlated with age (R = −0.149; *p* = 0.014). 

PTH levels were not associated with pre-transfusion hemoglobin (R = 0.129; *p* = 0.104) or ferritin levels (R = 0.065; *p* = 0.413) but were inversely associated with vitamin D levels (R = −0231; *p* < 0.0001).

No association was detected between PTH levels and MRI LIC values (R = 0.052; *p* = 0.396), global pancreas T2* values (R = −0.058; *p* = 0.343), global heart T2* values (R = −0.042; *p* = 0.490), or the number of segments with T2* < 20 ms (R = 0.045; *p* = 0.463).

PTH levels were comparable between patients without and with replacement myocardial fibrosis (17.76 ± 7.80 pg/mL vs. 17.42 ± 6.49 pg/mL; *p* = 0.965) and were uncorrelated with all biventricular function parameters (*p* > 0.05 in all correlations). 

### 3.4. Determinants of Global Heart T2* Values

The results of the stepwise regression analysis, including all significant variables in the univariate regression analysis with global heart T2* as the dependent variable, are shown in Table 3. Vitamin D, serum ferritin, and pancreatic iron levels were the strongest predictors of global heart T2* values (F = 20.65; *p* < 0.0001). No variable was excluded from the multivariable models due to excessive collinearity.

### 3.5. Best Cut-Off of Vitamin D for Cardiac Iron 

**Figure 2:**
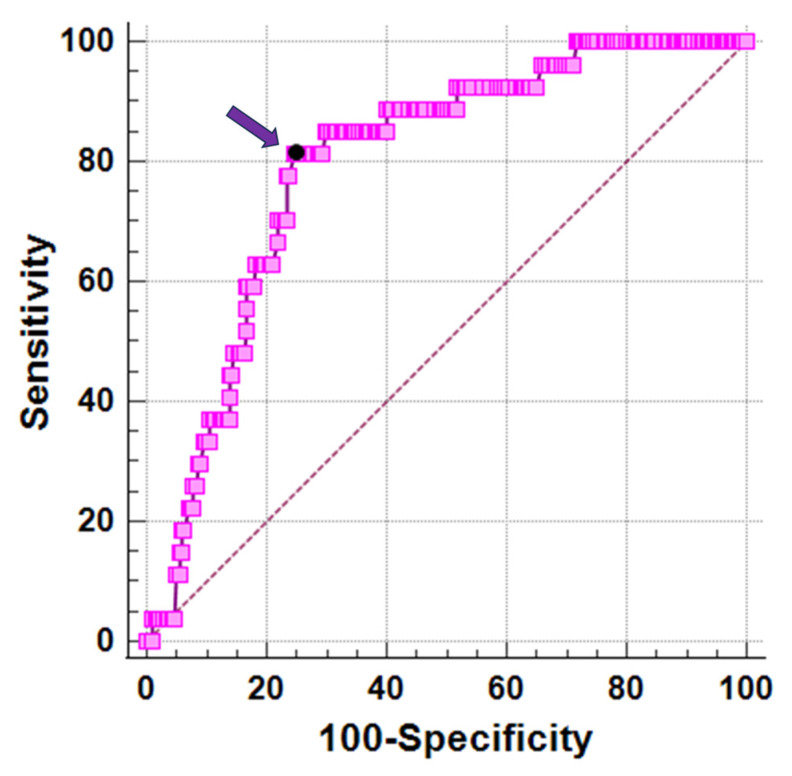
ROC curve analysis of vitamin D levels to predict myocardial iron overload. The arrow indicates the optimal cut-off value.

In receiver operating characteristic curve analysis, a vitamin D level ≤ 17.3 ng/dL predicted significant myocardial iron overload with a sensitivity of 81.5% and a specificity of 75.3% (*p* < 0.0001). The area under the curve was 0.79 (95%CI = 0.74–0.84) (Figure 2).

## 4. Discussion

We explored the cross-sectional association of vitamin D and PTH levels with pancreatic and cardiac iron and function in well-treated β-TM patients. 

We measured levels of 25(OH)D, which represented the best indicator of vitamin D status [59]. In total, 38.5% of our adult patients showed vitamin D deficiency. In a recent systematic review including twelve studies, the prevalence of vitamin D deficiency ranged from 24.8 to 80.6% [60]. The high discrepancy in the vitamin D deficiency prevalence among the published studies can be attributed to the differences in the cut-offs used for the definition of vitamin D deficiency, in the average age of the considered populations, and in the geographical areas, and, as a consequence, in sun exposure and in the protocols for vitamin D supplementation (type of supplement provided and durations/dosages of the intervention). Moreover, the timing of the blood sampling was not the same in all studies, and the vitamin D circulating in the bloodstream undergoes noticeable seasonal fluctuations, with the highest values seen in summer and autumn [61]. Importantly, it has been shown that, compared to sex- and age-matched healthy subjects, thalassemia patients have lower vitamin D levels [62,63,64]. Vitamin D deficiency in thalassemia patients has a multifaceted nature, involving inadequate dietary intake and absorption in the gastrointestinal tract, decreased skin synthesis of 25(OH)D3 due to jaundice or increased iron deposition in the skin, and impaired conversion of vitamin D to its active form (25-hydroxylation) due to iron-induced dysfunction in the liver [65,66,67]. The liver plays a fundamental role in metabolizing vitamin D, and disruptions in this process can contribute to deficiency. Indeed, in our study, hepatic iron overload was associated with an increased risk of inadequate vitamin D levels. This finding is consistent with previous studies aimed at assessing vitamin D insufficiency and its risk factors in patients with β-thalassemia [68,69].

We detected a significant association between pancreatic T2* values and vitamin D levels. However, the frequency of pancreatic iron overload was comparable among patients with sufficient, insufficient, and deficient vitamin D levels. This finding can be attributed to the high proportion of patients with pancreatic siderosis (93.2%), which makes it difficult to detect a difference among the three groups. 

Our study proved an association between decreased vitamin D levels and increased cardiac hemosiderosis, in accordance with the findings of Wood et al. [33] and Saadatifar et al. [70]. According to our data, patients with vitamin D deficiency faced a risk of myocardial iron overload 20 times higher than that of patients with adequate vitamin D levels and 8 times higher than that of patients with insufficient vitamin D levels. Importantly, in our multivariate regression analysis, vitamin D levels emerged as an independent predictor of global heart T2* values, suggesting that, besides pancreatic T2* measurements, the vitamin D level measurements should also be incorporated into clinical practice as risk stratification tools for myocardial iron overload. We introduced a vitamin D cut-off of 17.3 ng/dL for the identification of patients at increased high risk for myocardial iron overload. In these patients, it would be prudent to initiate or intensify vitamin D supplementation to prevent cardiac iron accumulation prospectively. We showed for the first time ever that the association between myocardial siderosis and low vitamin D levels was significant also considering a myocardial segmental analysis.

Vitamin D deficiency was associated with increased LV volume and mass. The association between vitamin D levels and LV EF did not reach statistical significance, likely due to the fact that the majority of our patients presented with normal or mild abnormal LV EF values. In TM, vitamin D can affect myocardial size and function not only indirectly by influencing cardiac iron uptake but also directly. The direct effects of vitamin D on the myocardium include a reduction in cardiomyocyte hypertrophy by the downregulation of specific genes [71], modulation of the renin–angiotensin system (RAS), which plays a key role in the regulation of volume and blood pressure homeostasis [72,73], moderation of extracellular matrix production and deposition in myocardial tissue [74], nongenomic and genomic influences on cardiac contractility and intracellular calcium regulation, and regulation of myosin expression and heart energy metabolism [75,76].

Although low levels of vitamin D can lead to an increase in the production of PTH, none of our patients exhibited increased PTH levels. Conversely, a small group of patients had reduced PTH levels. In TM, hypoparathyroidism is a rare and late complication caused by iron deposition in the parathyroid glands [77]. The low frequency of hypoparathyroidism in our population was mainly related to the optimal chelation therapy, which reduces the incidence of iron-related organ damage. The fact that most of our patients had normal PTH levels is the most likely explanation for the absence of a correlation between PTH and hepatic, pancreatic, and cardiac iron levels. Our finding is in line with a previous study on a smaller cohort of TM patients (*n* = 40) from Iran [41]. Compared to our study, the study which demonstrated a significant association between PTH and cardiac iron levels was characterized by a smaller patient size (62 vs. 278), a younger age of the involved patients (22.79 ± 6.18 years vs. 39.04 ± 8.58 years), and higher PTH levels (33.84 ± 16.72 pg/mL vs. 17.13 ± 7.66 pg/mL) [40].

### Limitations

The main limitation of this study is its cross-sectional design, with all data being collected at a specific point in time. Longitudinal studies are needed to establish a stronger causal relationship between vitamin D deficiency and increased cardiac iron uptake and to assess if adequate vitamin D supplementation can affect cardiac siderosis and function.

The data collection for the study did not include specific details regarding the type of supplement administered, such as the exact chemical form and the durations/dosages of the intervention.

## 5. Conclusions

In β-TM, vitamin D insufficiency/deficiency was common and associated with hepatic iron overload. Vitamin D deficiency emerged as a risk factor for myocardial iron overload, suggesting that the integration of vitamin D measurements into clinical practice may help to risk-stratify patients.

## Figures and Tables

**Table 1:** Demographic, clinical, laboratory, and MRI characteristics of β-TM patients.

β-TM Patients (*n* = 278)	
Age (years)	39.04 ± 8.58
Females, *n* (%)	158 (56.8)
Age at start of regular transfusions (months)	17.87 ± 16.97
Chelation starting age (years)	4.41 ± 4.69
BMI (kg/m^2^)	22.81 ± 3.37
Mean pre-transfusion hemoglobin (g/dL)	9.69 ± 0.50
Mean ferritin (ng/L)	1086.75 ± 1252.93
Vitamin D levels (ng/dL)	23.73 ± 10.90
Parathyroid hormone (pg/mL)	17.13 ± 7.66
Alanine aminotransferase (U/L)	30.34 ± 24.41
Aspartate aminotransferase (U/L)	28.33 ± 19.15
Gamma-glutamyl transferase (U/L)	25.81 ± 23.31
MRI LIC (mg/g dw)	7.38 ± 11.06
Hepatic iron overload, *n* (%)	145 (52.2)
Global pancreas T2* values (ms)	10.68 ± 8.39
Pancreatic iron overload, *n* (%)	259 (93.2)
Global heart T2* values (ms)	36.96 ± 10.11
Myocardial iron overload, *n* (%)	27 (9.7)
Number of segments with T2* < 20 ms	1.78 ± 4.46
LV EDVI (mL/m^2^)	84.70 ± 17.94
LV mass index (g/m^2^)	60.54 ± 13.18
LV EF (%)	63.46 ± 6.90
RV EDVI (mL/m^2^)	84.92 ± 20.74
RV EF (%)	61.54 ± 6.96
Replacement myocardial fibrosis, *n* (%)	77/191 (40.3)

TM = thalassemia major, *n* = number, BMI = body mass index, MRI = magnetic resonance imaging, LIC = liver iron overload, LV = left ventricular, EDVI = end-diastolic volume index, EF = ejection fraction, RV = right ventricular.

**Table 2:** Comparison of demographic, clinical, laboratory, and MRI findings among the three groups identified based on vitamin D levels.

	Normal Vitamin D (*n* = 75)	Insufficient Vitamin D (*n* = 96)	Deficient Vitamin D (*n* = 107)	*p*-Value
Age (years)	41.26 ± 8.19	39.63 ± 9.39	36.96 ± 7.64	0.001
Females, *n* (%)	47 (62.7)	59 (61.5)	52 (48.6)	0.089
BMI (kg/m^2^)	22.89 ± 3.41	23.05 ± 3.47	22.54 ± 3.25	0.538
Mean pre-transfusion hemoglobin (g/dL)	9.71 ± 0.40	9.74 ± 0.43	9.62 ± 0.64	0.411
Mean ferritin (ng/L)	797.08 ± 765.10	1014.99 ± 788.01	1425.75 ± 1822.79	0.049
Alanine aminotransferase (U/L)	23.49 ± 13.26	28.17 ± 20.80	36.59 ± 30.64	0.018
Aspartate aminotransferase (U/L)	24.12 ± 12.67	25.31 ± 15.35	33.45 ± 23.79	0.016
Gamma-glutamyl transferase (U/L)	22.50 ± 19.46	20.38 ± 14.94	31.06 ± 28.16	0.055
MRI LIC (mg/g dw)	3.68 ± 4.58	6.10 ± 6.62	11.11 ± 15.51	<0.0001
Hepatic iron overload, *n* (%)	23 (30.7)	49 (51.0)	73 (68.2)	<0.0001
Global pancreas T2* values (ms)	12.69 ± 7.65	11.03 ± 9.03	8.97 ± 8.01	<0.0001
Pancreatic iron overload, *n* (%)	71 (94.7)	88 (91.7)	100 (93.5)	0.734
Global heart T2* values (ms)	40.92 ± 5.96	38.25 ± 7.76	33.03 ± 12.66	<0.0001
Myocardial iron overload, *n* (%)	1 (1.3)	3 (3.1)	23 (21.5)	<0.0001
Number of segments with T2* < 20 ms	0.32 ± 1.56	0.89 ± 2.82	3.59 ± 6.14	<0.0001
LV EDVI (mL/m^2^)	79.17 ± 14.27	85.54 ± 18.32	87.61 ± 19.19	0.003
LV mass index (g/m^2^)	57.45 ± 12.90	59.84 ± 12.47	63.39 ± 13.54	0.005
LV EF (%)	64.41 ± 6.74	63.45 ± 6.79	62.77 ± 7.11	0.360
RV EDVI (mL/m^2^)	81.99 ± 21.97	84.18 ± 18.55	87.67 ± 21.60	0.056
RV EF (%)	61.38 ± 7.68	62.49 ± 6.77	60.79 ± 6.55	0.275
Replacement myocardial fibrosis, *n* (%)	24/49 (49.0)	25/64 (39.1)	28/78 (35.9)	0.332

*n* = number, BMI = body mass index, MRI = magnetic resonance imaging, LIC = liver iron overload, LV = left ventricular, EDVI = end-diastolic volume index, EF = ejection fraction, RV = right ventricular.

**Table 3:** Univariate and multivariate regression analysis for predicting global heart T2* values.

	Univariate		Multivariate	
	β	*p*-Value	β	*p*-Value
Female sex	0.025	0.679		
Age	0.189	0.002		
Pre-transfusion hemoglobin	−0.141	0.072		
Serum ferritin	−0.431	<0.0001	−0.344	<0.0001
Vitamin D levels	0.315	<0.0001	0.168	0.018
Parathyroid hormone	−0.053	0.387		
MRI LIC	−0.428	<0.0001		
Global pancreas T2*	0.316	<0.0001	0.241	0.001

MRI = magnetic resonance imaging, LIC = liver iron overload.

## Data Availability

The data presented in this study are available on request from the corresponding author. The data are not publicly available due to privacy concerns.
